# Infant Mortality

**Published:** 1875-01

**Authors:** 


### INFANT MORTALITY.

Prof. Henry Hartshorne read a valuable paper on this topic before the Public Health Association, at its last meeting, in which he expressed the justifiable opinion that among those born with a normal constitution, and under entirely favorable circumstances, the mortality during infancy and childhood ought to be less than at any other period of life. Yet it is a fact familiar to every one that the reverse is the case in very many localities—most notably in large cities. In France, according to Bouchut, one-sixth of all born die in the first year of life (Bertillon recently puts it at one-fifth); in Sweden and Finland, one-fifth; in Berlin, Prussia, one-third. Nor is the proportion very much less in some parts of England and this country. Before the war it was worst of all in New Orleans. In 1872, 1 death in 4½ occurred under one year of age in that city. The rate is declining somewhat during the last few years in Philadelphia and New York. From 1860 to 1872, the deaths under five years were 44.78 per cent., and under one year 27.25 per cent of the total mortality of Philadelphia. As Dr. Elisha Harris has remarked, Summer is the *tentator infantum* in New York. In the Summer quarter of 1868 in this city, the whole number of deaths being somewhat less than 8,700, of these nearly 5,600 were of children under 5 years of age; almost all being from what are called "diarrheal diseases." During one hot week of the Summer of 1870, three-fifths of the aggregate mortality in New York (645 deaths out of 1,048) occurred in children under five years; 400 deaths being from cholera infantum alone. In the hottest week of 1872, in Philadelphia, 852 deaths occurred, of which 497 were of infants under two years, 383 under one year; mostly from diarrheal disorders. The week previous to this gave 1,569 for the total mortality of New York, increased largely by the same mode of causation; such an aggregate of deaths probably having never been exceeded in this city; as the former (852) never has been in Philadelphia.

But the excessive mortality of early life is by no means accounted for by seasonal influences alone; other causes, also, are of great importance. These may be advantageously referred to as ante-natal and post-natal causes. Under the former head belong constitutional defects in parents. Among the causes of early mortality acting through parents, is excess of the nervous temperament and deficiency of organic development in women. It might be safer to say in men and women. Both run to brains and nerve too much in this country. Animal functions are less readily subordinated to the intellectual and moral nature, but all these rob too largely the vegetative, nutritive, and reproductive systems. This is the secret of the lessened and lessening number of births of American children of native parents, compared with those of foreign parentage. Much more remains to be investigated upon this subject, notwithstanding the elaborate inquiries of Dr. Allen, Dr. J. Stockton Hough, and others. In Massachusetts, at least, the mortality of infancy is greatest among the children of foreigners.

Post-natal causes of infantile mortality differ in different climates. Northern cities lose many infants in the Winter by pneumonia, capillary bronchitis, and croup—under the exposure to cold so often connected with poverty and neglect. Dr. Farr has shown that in London the degree to which the thermometer descends in December, January, or February, determines to a great extent the mortality of the winter.—Nothing in our mortuary statistics in

Philadelphia and New York is more constant than the proportion between the number of deaths among young children and the excess of the daily temperature above 95° Fahrenheit in the shade; indeed, we might safely say, about 90°. But, along with this positive cause of disease, taking effect most severely upon the infant population, must be apprehended and remembered also the action of impurity of atmosphere. Every zymotic disease is rendered more fatal, if not more prevalent, by foul air. Any sanitarian might designate in a city what wards, blocks, courts, alleys, and houses will always afford the largest number of deaths from scarlet fever, measles, and cholera infantum from year to year, and from diphtheria, cerebro-spinal fever, typhus, or cholera, when either of these prevail. The great importance of impurity of the atmosphere as a factor in the mortality of infants has been fully recognized in large cities in times past. There seems to be some ground for fear that it may be, at the present time, too little borne in mind, under the almost overshadowing attention given to another factor, itself truly of great conseqence—bad feeding of children. Errors in infantile diet may be considered briefly, as they occur: First, when the child is suckled, in part or altogether, by the mother or a substitute; and, second, when it is fed entirely by hand or with the bottle. Feeble mothers cannot often, although they do sometimes, rear healthy children. Women obliged to work hard, and sometimes to leave their infants for many hours together, neglect them, almost or quite unavoidably, to a great disadvantage. Weaning occurs thus prematurely, and privation of natural food invites early death. At the opposite scale of society, in some countries, most of all in France, but to a small extent only in America, indolence and luxury among the rich induce mothers to thwart the instinct of maternity by placing their offspring under the care of hireling nurses, often far away from their homes. The large mortality of children so treated has for a number of years past attracted the serious attention of French physicians and sanitary observers. Bertillon reckons that one-half of the nurse-children of Paris perish during the first year. The same sort of evil is intensified fearfully in foundling hospitals, whose death-rate has always been immense.

In the care of infants the worst errors are, first, giving infants stale milk; second, watering the milk overmuch; third, substituting farinaceous or other food, incompetent to supply tissue-waste and maintain life. To lessen the influences hostile to infantile life and health we must look chiefly to popular education, moral reform, and sanitary police. Under the last named should be included inspection and sanitary improvement of dwellings and localities in cities. Means should be taken to diffuse information among all classes, and especially the poor, concerning food most of all the need of freshness and purity in that which is given to children), cleanliness, and ventilation. Holly-tree inns and temperance coffee houses ought to be established, to give cheer and comfort, without inebriation, in every quarter of every city. Children's excursions in hot weather should be, as they now are, made the generous duty of the richer, and the life-giving enjoyment of the poorer class. Summer camps for mothers with young infants, during hot weather, should be provided outside of every large city. Such camps would be training-schools in healthy living to all who occupied them, the effects of which would last long afterwards. Moreover, by the removal of a part of their population, the worst quarters of the cities so relieved might be open to inspection, and effectual, permanent, compulsory sanitation.

